# Supplementary material for: The Clinical Value of Pre-Diagnostic Thrombocytosis for the Detection of Lung Cancer in Primary Care
Source: Cancers (Basel). 2024 Mar 14;16(6):1154. doi: 10.3390/cancers16061154 (PMC10969421; doi:10.3390/cancers16061154)
Supplement: Supplementary file 1 [file cancers-16-01154-s001.zip › Table S2.pdf]

Table S2: Conditional logistic regression for the predicted odds ratios and incidence of pre-diagnostic thrombocytosis occurrence, clustering by case-control matching. Adjusted for significant predictors of pre-diagnostic thrombocytosis: smoking status, previous COPD diagnosis, and anti-platelet drug prescriptions.. * baseline variable, OR odds ratio, CI confidence interval.

	**Adj. OR**	**95% CI**	***p*-value**
Case-control			
Control	1*		
Case	8.9	8.0 to 9.9	<0.001
Smoking-status			
Never-smoker	1*		
Ever-smoker	1.5	1.2 to 1.8	0.001
Previous COPD diagnosis			
No	1*		
Yes	2.7	2.2 to 3.3	<0.001
Anti-platelet drug prescription			
No	1*		
Yes	1.2	1.0 to 1.4	<0.05
