# Supplementary material for: The Clinical Value of Pre-Diagnostic Thrombocytosis for the Detection of Lung Cancer in Primary Care
Source: Cancers (Basel). 2024 Mar 14;16(6):1154. doi: 10.3390/cancers16061154 (PMC10969421; doi:10.3390/cancers16061154)
Supplement: Supplementary file 1 [file cancers-16-01154-s001.zip › Table S3.pdf]

Table S3: Sensitivity analysis on patients with a pre-diagnostic platelet count record only. Incidence of pre-diagnostic thrombocytosis in cases and controls, by sex and histological subtype for patients with a pre-diagnostic platelet count record. ADC adenocarcinoma, SCC squamous cell carcinoma, SCLC small cell lung cancer, CI confidence interval.

	**All patients, % (95% CI)**		**Males, % (95% CI)**		**Females, % (95% CI)**	
	**Cases**	**Controls**	**Cases**	**Controls**	**Cases**	**Controls**
**All patients**	18.3 (17.4 – 19.2)	3.0 (2.7 to 3.2)	15.4 (14.2 to 16.6)	1.7 (1.5 to 2.0)	4.2 (3.9 to 4.7)	21.3 (20.0 to 22.8)
**ADC**	15.1 (13.7 – 16.7)	2.9 (2.5 to 3.3)	12.3 (10.5 to 14.4)	1.6 (1.2 to 2.1)	4.0 (3.4 to 4.7)	17.8 (15.6 to 20.1)
**SCC**	22.5 (20.4 – 24.7)	2.6 (2.2 to 3.1)	20.5 (18.0 to 23.3)	1.8 (1.3 to 2.3)	4.1 (3.2 to 5.1)	25.8 (22.3 to 29.6)
**SCLC**	16.0 (13.6 – 18.7)	2.7 (2.1 to 3.4)	14.2 (10.9 to 18.1)	1.8 (1.1 to 2.7)	3.5 (2.5 to 4.7)	17.6 (14.1 to 21.6)
