# Supplementary material for: The Clinical Value of Pre-Diagnostic Thrombocytosis for the Detection of Lung Cancer in Primary Care
Source: Cancers (Basel). 2024 Mar 14;16(6):1154. doi: 10.3390/cancers16061154 (PMC10969421; doi:10.3390/cancers16061154)
Supplement: Supplementary file 1 [file cancers-16-01154-s001.zip › Table S4.pdf]

Table S4: Conditional logistic regression for the predicted odds ratios and incidence of pre-diagnostic thrombocytosis occurrence for cases and controls, clustering by case-control matching identifier. Adjusted for significant predictors of pre-diagnostic thrombocytosis: smoking status, and previous COPD diagnosis. * baseline variable, OR odds ratio, CI confidence interval.

	**Adj. OR**	**95% CI**	***p*-value**
Case-control			
Control	1*		
Case	6.3	5.6 to 7.2	<0.001
Smoking-status			
Never-smoker	1*		
Ever-smoker	1.3	1.0 to 1.6	<0.05
Previous COPD diagnosis			
No	1*		
Yes	1.6	1.3 to 2.0	<0.001
